# Mango Pectic Oligosaccharides: A Novel Prebiotic for Functional Food

**DOI:** 10.3389/fnut.2022.798543

**Published:** 2022-03-24

**Authors:** Malaiporn Wongkaew, Pipat Tangjaidee, Noppol Leksawasdi, Kittisak Jantanasakulwong, Pornchai Rachtanapun, Phisit Seesuriyachan, Yuthana Phimolsiripol, Thanongsak Chaiyaso, Warintorn Ruksiriwanich, Pensak Jantrawut, Sarana Rose Sommano

**Affiliations:** ^1^Program in Food Production and Innovation, College of Integrated Science and Technology, Rajamangala University of Technology Lanna, Chiang Mai, Thailand; ^2^Plant Bioactive Compound Laboratory, Faculty of Agriculture, Chiang Mai University, Chiang Mai, Thailand; ^3^Faculty of Agro-Industry, School of Agro-Industry, Chiang Mai University, Chiang Mai, Thailand; ^4^Cluster of Agro Bio-Circular-Green Industry (Agro BCG), Chiang Mai University, Chiang Mai, Thailand; ^5^Department of Pharmaceutical Sciences, Faculty of Pharmacy, Chiang Mai University, Chiang Mai, Thailand; ^6^Cluster of Research and Development of Pharmaceutical and Natural Products Innovation for Human or Animal, Chiang Mai University, Chiang Mai, Thailand; ^7^Department of Plant and Soil Sciences, Faculty of Agriculture, Chiang Mai University, Chiang Mai, Thailand

**Keywords:** fruit biomass, intestinal microflora, lactic acid bacteria, short-chain fatty acids, probiotic

## Abstract

Prebiotics are functional food ingredients that assist probiotic growth and render many other health benefits. Mango peel is the biomass of the processing industry and has recently been value-added as a dietary fiber pectin. Besides its general use as a food additive, mango peel pectin (MPP) is partially hydrolyzed by pectinase to obtain pectic oligosaccharides (POSs) that have recently gained attention as novel prebiotic products and in medical research. This review describes probiotic candidates responsible for the digestion of pectin derivatives and the advantages of POSs as functional additives and their current best retrieval options. Mango pectic oligosaccharide (MPOS) recovery from low methoxyl MPP from mango with prebiotic performance both *in vivo* and *in vitro* environments is discussed. Current research gaps and potential developments in the field are also explored. The overall worthiness of this article is the potential use of the cheap-green food processing bioresource for high-value components.

## Introduction

Functional food is a type of food that is supplemented with bioactive ingredients (e.g., dietary fiber, probiotics, and antioxidants) and derived food ingredients. It can be consumed as part of a normal daily diet that provides health benefits and reduced the risk of chronic diseases beyond those provided by adequate nutrition. The newborns' gastrointestinal (GI) tracts were inoculated with organisms at an early stage of life due to the influence of maternal intestinal flora and diets (1). These include aerobic Gram-positive cocci, enterobacteria, and *Lactobacilli*, which are the primary colonizers. These bacteria rapidly consume O_2_, which enhances the growth of obligate anaerobic species, collectively known as gut microflora (2, 3). In particular, the microflora of breast-fed infants is dominated by a bifidobacterial population that purports to be in a better health condition than formula-fed babies (4). Human milk oligosaccharides (HMOs) are enriched with complex glycan compounds that are partially mediated by the modulation of the intestinal microbial ecology and immunological homeostasis associated with the prevention of intestinal diseases, improved general wellbeing, and reduced incidence of allergic symptoms (5, 6). Consequently, galacto-oligosaccharides (GOSs) are often the predominant prebiotic oligosaccharides used in infant diets. It is believed that including GOSs in infant formula boosts the population of bifidobacterial, reduces pathogenic inoculums, and boosts metabolic activity in immune system regulation (7). GOSs are typically synthesized from lactose through the enzymatic activity of β-galactosidase (as well as β-glucosidases and β-glycosidases) *via* transgalactosylation (8). Besides GOSs, other prebiotic oligosaccharides such as fructo-oligosaccharide (FOS) and/or polydextrose (PDX) are also prevalent components in breast milk, but they are fundamentally absent in cow's milk (9–11). In addition to the ability to enhance the growth of *Bifidobacteria* and *Lactobacilli*, short-chain fatty acids (SCFAs) are produced as the by-products of oligosaccharide fermentation. In adult humans, the SCFA has a stronger link in the prevention of colon cancer (12). Recently, POS has been proposed as a new class of prebiotics capable of *in vivo* synergistic empowerment of immunomodulation caused by GOS and FOS (13, 14). Mango peel is a potential biomass for dietary fiber recovery with 5–11% pectin depending on the extraction methods, varieties, and, also, fruit morphological characteristics (15, 16). As a food additive, mango peel pectin (MPP) has been utilized as a food additive to alter the texture and firmness of food products, and a carrier material for drugs and medicine (17, 18). The extracted MPP could be partially hydrolyzed by a pectinase enzyme to obtain MPOS as a prebiotic in human food (19).

## Probiotics

Probiotics are defined by the World Health Organization as live microorganisms that enhance health benefits on the host when consumed in adequate amounts (20). They are non-pathogenic, beneficial, and active bacteria and yeast. The highly potent and commonly used probiotics are *Lactobacillus* spp., *Bifidobacterium* spp., *Saccharomyces boulardii, Propionibacterium* spp., *Streptococcus* spp., *Bacillus* spp., *Enterococcus* spp., and some specific strains of *Escherichia coli* (Table 1). Especially, the genus of *Lactobacillus* and *Bifidobacterium* of the Gram-positive, non-spore-forming, non-mobile, obligated are those of the facultative anaerobic bacteria. They are classified as lactic acid bacteria (LAB), which are catalase-negative bacterial species that can produce lactic acid as the main end-product of carbohydrate fermentation (23). LAB are generally used as food additives (Table 2) for health-promoting purposes.

**Table 1:** Most important representatives of probiotic microorganisms.

***Lactobacillus* species**	***Bifidobacterium* species**	**Other LABs**	**Non-LABs**
*L. acidophilus*	*B. adolescentis*	*Enterococcusfaecalis* ^a^	*Bacillus cereus* var. to yoi^a^
*L. casei*	*B. animalis*	*E. faecium*	*Escherichia coli* strain nissle
*L. crispatus*	*B. bifidum*	*Lactococcus lactis*	*Propionibacterium freudenreichii*
*L*.*galinarum*^a^	*B. breve*	*Leuconostoc mesenteroides*	*Saccharomyces cerevisiae*
*L. gasseri*	*B. infantis*	*Pediococcus acidilactici*	*S. boulardii*
*L. johnsonii*	*B*.*lactis*^b^	*Sporolactobacillus inulinus*	
*L. paracasei*	*B. longum*	*Streptococcus thermophilus*	
*L. plantarum*			
*L. reuteri*			
*L. rhamnosus*			

a *Mainly used for animals*.

b *Recently reclassified as Bifidobacterium animalis sub sp. lactis (21)*.

*Adapted from Holzapfel et al. (22) and Kechagia et al. (23)*.

**Table 2:** Probiotic products and their compositions.

**Products**	**Probiotic compositions**
Align	*B. infantis* 35,624; 4 mg/capsule = 1 billion CFU
Activia yogurt	*B. lactis*; 100 million bacteria per g
Culturelle	*L. rhamnosus*: 10 billion bacteria plus insulin 200 mg per capsule
Culturelle for kids	1.5 billion bacteria per packet
Howaru	*L. acidophillus*/*B. lactis*: 10 billion bacteria per capsule
Kefir	*L. lactis, L. rhamnosus, L. plantarum, L. casei, L. acidophillus, L. reuteri, Leuconostoc cremosis, Streptococcus diacetylactis, S. florentinus, B. longum, B. breve, B. lactis*: 7–10 billion CFU per cup
Lactinex	*L. acidophilus, L. bulgaricus*: 10^6^ CFU/tablet and 10^9^ CFU/packet
Protectis	*L. reuteri*: 100 million bacteria per dose
RepHresh Pro-B	*L. rhamnosus, L. reuteri*: 5 billion CFU per capsule; vaginal use
VSL#3	*L. plantarum, L. paracasei, L. bulgaricus, B. breve, B. infantis, B. longum, S. thermophiles*: 225 billion bacteria per 2 capsules
Yakult	*L. casei*: 8 billion bacteria per 80 mL bottle

*CFU, colony forming unit*.

*Pharmacist's Letter 2012; 28(7):280707. Islam (24)*.

**Figure 1:**
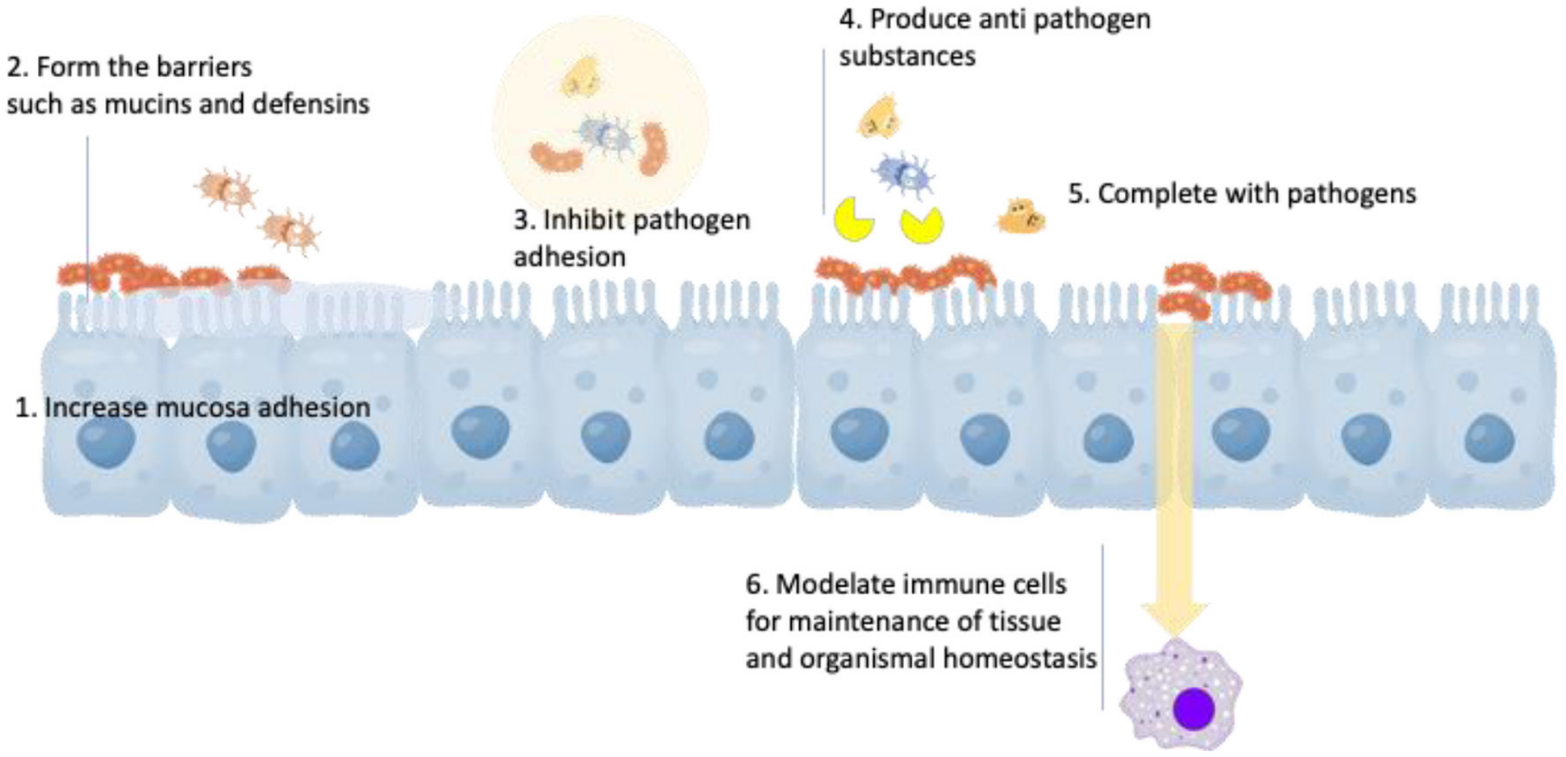
Major mechanisms of action of probiotics.

The health mechanisms of these probiotic genera are described in Figure 1, which includes (1) increased adhesion to the intestinal mucosa, (2) enhancement of the epithelial barrier, (3) inhibition of pathogen adhesion, (4) production of antimicroorganism substance, (5) competitive exclusion of pathogenic microorganisms (i.e., acid and SCFA), and (6) modulation of the immune system (25, 26). *Lactobacillus reuteri* is a well-studied probiotic bacterium that colonizes a large number of mammals. It has been clarified as a heterofermentative species that can grow in oxygen-limited atmospheres and colonize the GI tract of humans and animals (27). It can also survive in a variety of pH conditions, employ multiple mechanisms for pathogenic microorganism inhibition, and secrete many antimicrobial intermediates (28–30). *B. animalis* is considered a natural inhibitor of human and other mammalian GI tracts, as well as widely supplemented in numerous fermented dairy products (31). The major subspecies of *B. animalis* include *animalis* and *lactis*, of which the latter subspecies is regarded as technologically suitable to use as a probiotic adjunct due to its resistance against acid, bile, and oxygen than other members of the genus (32, 33). Clinical properties for health advancement and/or disease defense of the probiotics are to provide symptom relief to individuals with common GI symptoms, irritable bowel syndrome, and constipation and to boost the immune response (34). The survival rate of probiotics in gastric conditions depends on the types of prebiotics and their resources. Larsen et al. (35) claimed that *Lactobacillus fermentum* PCC and *L. reuteri* RC-14 were more resistant to gastric tract in the presence of different pectin types. The variable amount of polygalacturonic acids in the backbone was also crucial for bacterial protection (35). The protective function of pectin is linked primarily to the complex fluctuation in surface charges and, as a result, influences the pectin-bacteria electrostatic interactions. Additionally, Corcoran et al. (36) and Hernandez-Hernandez et al. (37) suggested that the survival improvement of *Lactobacilli* in the gastric juice by prebiotic oligosaccharides was in association with the presence of metabolizable sugars, which could maintain pH homeostasis by increasing ATP generation.

## Types of Prebiotics as Functional Food Ingredients

Prebiotics are short-chain carbohydrates (SCCs) that are non-digestible by digestive enzymes in humans and sometimes known as resistant SCCs because they are only fermented in the intestinal tract (38). It encourages the growth and/or activity of one or a limited number of intestinal bacteria that reside in the gut rather than introducing the exogenous species (39). Non-digested carbohydrate (CHO) molecules, saccharides (di-, oligo-, and poly-), resistant starches, and sugar polyols have been claimed to have the potentiality of prebiotic (40). The carbohydrate sources are identified as prebiotic properties that achieve the following criteria; (a) resistance to gastric acidity and mammalian enzymes, (b) susceptibility to fermentation by gut bacteria, and (c) ability to enhance the viability and/or activity of beneficial microorganisms (41). For these, GOS, FOS, and inulin are commercially accepted for food-grade prebiotics. GOSs and FOSs are non-digestible carbohydrates derived from lactose that can be found naturally in human milk. Inulin and fructan are known as prebiotics derived from soluble dietary fibers, which are vastly obtained from plants such as asparagus, chicory, tomatoes, mango, onion, and garlic (40, 42). Prebiotic compounds are classified based on chemical structures, chain length or degree of polymerization, and applications (43). Most of the functional food prebiotics are saccharide derivatives, which are mostly of plant origin. Besides, protein or peptide and lipid prebiotics can be naturally found (44). The different types of prebiotics and their plant sources are summarized in Table 3. Inulin is a polysaccharide fructan that produces SCFAs that are extracted from fruits and vegetables. The fatty acids such as propionate, butyrate, and acetate endure the reduction effect of lipids and cholesterol and possibly attain a reduction in hypertension risk (58). Similarly, pectic oligosaccharides (POSs), which are the products of partial hydrolysis of pectin, are currently classified as emerging prebiotics, but only limited studies are there to support their use at the commercial level (59).

**Table 3:** Types and sources of natural prebiotics.

**Prebiotic types**	**Prebiotic sources**	**References**
Arabinoxylooligosaccharides	Wheat bran	(45)
Cyclodextrins	Water-soluble glucans	(46)
Enzyme-resistant dextrin	Potato starch	(47)
Fructooligosaccharides	Asparagus, sugar beet, garlic, chicory, onion, Jerusalem artichoke, wheat, honey, banana, barley tomato, rye	(48)
Galactooligosaccharides	Human's milk and cow's milk	(49)
Isomaltulose	Honey, sugarcane juice	(50)
Isomaltooligosaccharides	Starch	(51)
Lactulose	Lactose (milk)	(52)
Maltooligosaccharides	Starch	(51)
Pectic oligosaccharide	Mango, sugar beet, citrus	(19, 53, 54)
Raffinose oligosaccharides	Seeds of legumes, lentils, peas, beans, chickpeas, mallow composite, mustard	(55)
Soybean oligosaccharide	Soybean	(56)
Xylooligosaccharides	Bamboo shoots, fruits, vegetables, milk, honey, wheat bran	(57)

## Pectic Oligosaccharides

Pectic oligosaccharide is a non-digestible oligosaccharide that possesses prebiotic activity. POS beneficially affects the host by selectively enhancing the growth and/or activity of one or a limited number of *Bifidobacteria* and *Lactobacilli* in the colon (56, 60). The colonic fermentation of POS generates SCFA, which provides a great variety of health effects, including inhibition of pathogenic bacteria, constipation relief, reduction in blood glucose levels, improvement in mineral absorption, reduction of colonic cancer, and modulation of the immune system (13). POS also has a potential inhibitory on the growth of entero-putrefactive and pathogenic organisms (61, 62). Pectic polysaccharide (i.e., pectin) extracted from various sources is cut into smaller chains of POS using different preparations, *viz*. enzymatic, chemical, and physical techniques (63, 64). The techniques for POS preparation from several raw materials and biomasses are comprehensively collected as shown in Table 4. Enzymatic treatment has been extensively applied for POS production due to the specificity and selectivity as well as minimum adverse chemical modifications of products (74, 75). The hydrolysis enzyme of pectin has been used, which acts synergistically to produce POS (64, 76, 77). The methyl esters of galacturonic acid residues are cleaved by pectin methyl esterase (PME) (78). This enzyme acts before polygalacturonase (PG). PG degrades the glycosidic bond of the α-(1,4)-polygalacturonan in a random position (79). Nevertheless, the less esterified the structure of the pectin substrate, the greater the activity of PG (80). Meanwhile, pectin lyase (PL) highly catalyzes the esterified pectin, producing unsaturated methyloligogalacturonates through transelimination of glycosidic linkages (81). Physical pretreatments, including hydrothermal, dynamic high-pressure microfluidization (DHPM), and irradiation, have been adapted to partially degrade the raw materials for oligosaccharide release (71, 82, 83). Using the hydrothermal method, arabino and galacto-oligosaccharides (GOSs) were effectively obtained from various bioresources. While the chemical hydrolysis of pectin for the production of POS has been limitedly explored. This is because there are some disadvantages to the chemical process, including toxicity and limitation of the desired degree of polymerization (75).

**Table 4:** Preparation of POS from various sources by different techniques.

**Sources/ types of POS**	**Preparation techniques**	**Molecular weight**	***In vitro* probiotic test**	**Major SCFAs products**	**References**
Apple pomace	Enzymatic technique (Pectinex Ultra SP-L, Viscozyme, Rohapect Ma Plus T, Rapidase Smart)	DP 7–10	*Lactobacillus plantarum, L. brevis, L. paracasei, Leuconostoc mesenteroides*	Acetic acid, propionic acid	(65)
Artichoke	Enzymatic technique (Peclyve CP)	0.3–100.0 kDa	n/a	n/a	(66)
Citrus peel	Enzymatic technique (Peclyve CP)	<1.0–1.8 kDa	*Bifidobacterium bifidum* and *L. acidophilus*	n/a	(67)
Lemon peel	Enzymatic technique (Crude gungal PL and yeast PG)	51.4 kDa	Bacterial groups; Bifidobacterium, *Lactobacillus*, Enterococcus	Acetic acid, butyric acid	(68)
Mango peel	Enzymatic technique (Pectinex® ultra tropical)	<1.0 kDa	*B. animalis* and *L. reuteri*	Acetic acid, propionic acid	(19)
Orange peel	Enzymatic technique (Fungal crude enzyme; pectinase, cellulase, CMCase, xylanase)	<1.0–>3.0 kDa	*B. infantis* and *L. acidophillus*	n/a	(69)
Sugar beet pulp	Enzymatic technique (Crude gungal PL and yeast PG)	63.9 kDa	Bacterial groups; *Bifidobacterium, Lactobacillus, Enterococcus*	Acetic acid, butyric acid	(68)
Sunflower	Enzymatic technique (Commercial cellulase *Aspergillus niger* with pectinase)	100–800 kDa	Bacterial groups from fecal	Acetic acid	(70)
Apple pectin	Physical technique (Dynamic high-pressure microfluidisation)	n/a	Bacterial groups from fecal	Acetic acid, propionic acid	(63)
Lemon peel waste	Physical technique (Hydrothermal treatment)	DP 2–18	n/a	n/a	(71)
*A. argute* fruit	Mixed technique (Ultrasound-assisted enzymatic treatment)	<0.7–>3.0 KDa	Bacterial groups from fecal	Acetic acid, propionic acid, butyric acid	(72)
Hawthorn fruit	Mixed technique (Ultrasound-assisted enzymatic treatment)	0.8–2.2 KDa	n/a	n/a	(73)
Citrus peel pectin	Chemical technique (Trifluoroacetic acid)	2.0–4.0 KDa	*Bifidobacterium bifidum* and *L. acidophilus*	n/a	(54)

*DP, degree of esterification; PL, pectin lyase, PG, polygalacturonase*.

**Figure 2:**
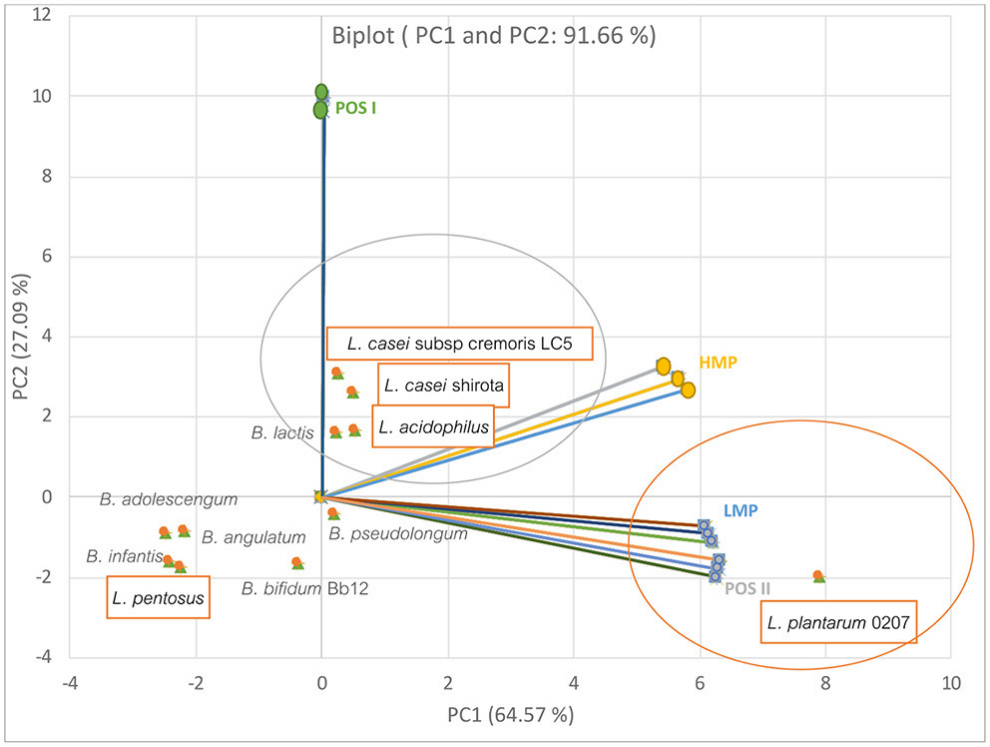
The relationship between prebiotic types and the specific growth rate of selected gut bacteria. The plot was regenerated from the data presented in (84).

Furthermore, probiotics in the gut system respond differently to alternate types of prebiotics. Olano-Martin et al. (84) reported that different types of pectins and POSs had significant selective effects on the growth of gut bacteria as shown Figure 2. The data were regenerated using the principal component analysis (PCA). From the figure, the first two dimensions of the PCA accounted for 91.66% across the PCA score plot (PC1; 64.57% and PC2; 27.09% of the variance). Overall, it was found that the *Lactobacillus* spp. gave mostly positive responses to the prebiotic pectins, while *Bifidobacteria* appeared opposingly. It was also apparent that the low methoxyl pectin (LMP) and its pectic oligosaccharide hydrolysate (POS II) were specifically responsive to *Lactobacillus plantarum* 0207. Meanwhile, high methoxyl type (HMP) and its hydrolysate (POS I) had a slight influence on the growth of the two strains of *Lactobacillus casei* and *Lactobacillus acidophilus*.

## Mango Pectic Oligosaccharides

### Purification

Mango peel accounts for 20% of fruit biomass and is known as a potential source of dietary fiber consisting of a high pectin (5–10%) composition depending on fruit characteristics, the extractions, and varieties (16, 37–41). Previous studies have illustrated that peel from the Thai mango variety, Chok Anan, provided a substantially high amount of pectin (13%), mainly of low methoxyl content, illustrating gelation properties at low sugar content; thus, it has been widely used as a food additive (18). The MPP was used as a potential source for POS II with pectinases and the longer the hydrolysis time and pectinase concentrations, the lower the molecular weight (M_w_) obtained (85). The MPOS was evaluated for prebiotic activity with *L. reuteri* and *B. animalis*, whose highest proliferation was 4% (w/v) of the MPOS supplemented condition at 72 h of the fermentation time (19). Pectinase and long hydrolysis cleaved pectin to be active molecules with high prebiotic efficiency (13). It is encouraged that purification processes are highly recommended to obtain food-grade final products. Generally, the purification of POS can be operated either by membrane-based technology, including those of ultrafiltration with the regenerated cellulose membrane of different pore sizes (83, 86), or chromatographic separation (87, 88) with a specific resin/matrix (74). The purification steps are chosen based on the selection of the components from the mixture. In the case of POS produced from orange peel wastes (OPWs), the POS was purified by a two-step membrane process (i.e., discontinuous diafiltration and concentration) to yield a refined product comprising up to 90% oligosaccharides (89). Likewise, Holck et al. (90) implemented a regenerated cellulose membrane for purification of sugar beet POS, while Iwasaki and Matsubara (91) purified the oligomers acquired from citrus pulp pectin from the membrane using two steps to maintain the high MW constituents and eliminate a small molecule of monosaccharides and saccharose.

### Structural Characterization

The POS structure is complicated because of the complex chemical composition of pectin and the chemical alteration during POS production. The complex mixtures of POS can be analyzed using high sensibility and ability methods such as matrix-assisted laser desorption ionization-mass spectrometry (MALDI-MS) (92), electrospray ionization (ESI)-MS (93), capillary electrophoresis-MS (94), capillary electrophoresis with UV detection (95), and NMR and ESI-MS identification (96–98) with fluorescent labeling (99). Arabino-oligosaccharides degraded from sugar beet pulp pectin were able to be classified by MALDI-time-of-flight-MS and high-performance anion exchange chromatography with pulsed amperometric detection (HPAEC-PAD) (100). The M_w_ of POS can be evaluated using the size exclusion chromatography (SEC) (19, 101), HPAEC-PAD (64), as well as hydrophilic interaction liquid chromatography (HILIC) with online ESI ion trap-MS-evaporative light scattering detection (ELSD) (102). The presence of Mw of MPOS affected the probiotic growth because the utilization of prebiotics by lactic acid requires the presence of specific enzyme hydrolysis and transport systems for the particular prebiotic (103). The β-galactosidase activity in the tested strains was correspondent with low-molecular-weight substrate (104). For the quantity analysis of sugars in POS liquefaction, high-performance liquid chromatography (HPLC) (19), HPAEC-PAD (53), and HPAEC-fluorescence detection (105) are widely used to specify the monosaccharide contents in the recovered POS. The major sugar compositions of the oligosaccharide from mango peel were fructose (24.41%) and glucose (19.52%) (19).

### *In vivo* and *in vitro* Performance

Pectic oligosaccharides have been proposed as a new class of prebiotics capable of exerting a number of health-promoting effects, including bifidogenic flora promotion, antioxidant activity (106), lowering the serum levels of total cholesterol and triglyceride (107), antiadhesive properties for food pathogen toxins (*E. coli* O157:H7), and apoptosis stimulation of colon cancer cells (108). For the simulation of prebiotic fermentation, *B. animalis* TISTR 2195 showed higher proliferation in 4% (w/v) of MPOS supplemented (8.92 log CFU/ml) than that of *L. reuteri* (8.53 CFU/ml) at 72 h of the fermentation time. This may be as a result of the intracellular enzymes of *Bifidobacterium*, which could hydrolyze the oligosaccharides into monosaccharides (i.e., glucose and fructose phosphates) and utilize them as a nutrient source (67). The main SCFAs derived from MPOS were acetic acid and propionic acid. Both acids are known as the main SCFAs derived from POS fermentation (109). The highest value of total SCFA was achieved from the 4% (w/v) MPOS supplementation for both *B. animalis* (68.57 mM) and *L. reuteri* (69.15 mM).

Besides, POS defends colonic cells against Shiga toxins (Stx) secreted from *E. coli* O157:H7 by neutralization of Stx activity from POS interaction with the galabiose receptor (110). The higher the molecular mass of POS, the greater the inhibitory activity obtained. This may be due to the increased access to the receptor-binding sites on the toxin. POS also prevents the adhesion of P-fimbriated *E. coli* to uroepithelial cells *in vitro*. Oligogalacturonic acid, disaccharide, and trisaccharide were the most active POS (111). In the case of cancer preventative ability, POS can stimulate the apoptosis process in human colonic adenocarcinoma cells (112). The incident is due predominantly to the growth enhancement of *Bifidobacteria* and further promotes their immunomodulatory capacity (113). *In vivo* synergistic empowerment of immunomodulation caused by GOSs and FOSs mixed with pectin-derived acidic oligosaccharides showed systemic Th1-dependent immune responses in a murine vaccination model. It can therefore be assumed that the application of these oligosaccharides in infant formulas is beneficial for the development of the infant's immune system (114). The potential for *in vivo* cardiovascular protection of POS is also reported by Li et al. (107), and it was found that haw POS (HPOS) significantly reduced the serum levels of total cholesterol and triglycerides and inhibited the accumulation of body fat. Therefore, HPOS can be applied as a drug therapy to combat cardiovascular diseases. Additionally, *in vivo* and *in vitro* studies confirmed that acidic POS was not cytotoxic or mutagenic in the Ames test, making it suitable for use in food products for children and babies (60).

## Clinical Practice Guidelines

**Figure 3:**
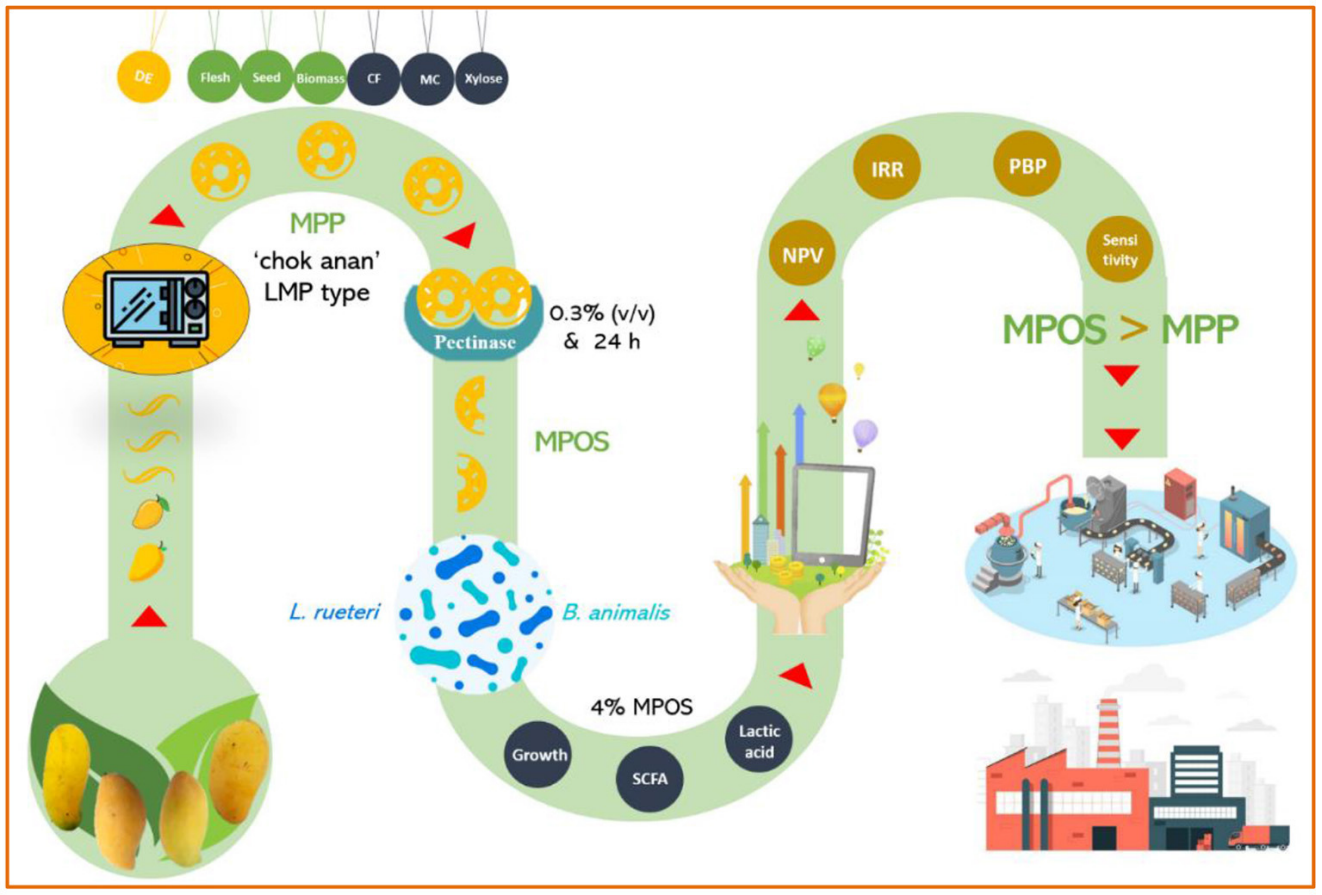
Mango peel pectin and mango pectic oligosaccharide recovery from different varieties of mango fruits.

Prebiotics are substances that exist naturally in food or are fortified during manufacturing to improve the functional efficacy of probiotics. Food containing both prebiotics and probiotics is usually recognized as “symbiotic” (43). The fructan inulin is the common prebiotic that has been categorized as “Generally Accepted as Safe” by the American Food and Drug Administration (58). POSs of plant origins have been proposed as excellent candidates for new-generation prebiotics (115–117). It is believed that microorganisms are able to utilize the carbon sources from POS better than they are with the polysaccharides, and in fact, pectinolytic enzymes are only characterized by *Bacteroides* sp. and *Clostridium butyricum-Clostridium beijerinckii* group in the human gut (84). The prebiotic effect of POS depends upon the M_w_ of the fractions. Low-molecular-weight POS has better prebiotic potential than high-molecular-weight POS (118), even though the clinical trials and toxicity studies are limited. POSs have also been shown to possess *in vitro* anti-inflammatory and antioxidant activity (66, 119). It also illustrates a vasoprotective effect that increased the serum SOD activity and lowered the content of MDA in these mice that are fed a high-fat diet, which can be used as a supplement for protection against cardiovascular diseases (107). Moreover, POS improves the gut mucosal structure, which is a barrier for rotavirus infection that induces diarrhea caused by damaging the intestinal organs in children and young animals (120). It is no doubt that MPOS is a novel prebiotic dietary fiber with antipathogenic potential against *Staphylococcus aureus, E. coli, Bacillus subtilis*, and *Salmonella typhimurium* (121). The research study regarding these novel prebiotic resources is in a very early stage. Optimization of the extraction and purification steps together with the characterization of POS derived from the MPP still needs to be explored to a greater extent. Consequently, the clinical studies and safety evaluation of MPOS used in food should be performed. All in all, it is conclusively recommended from the information gathered herein that MPOS is a novel functional food ingredient that provides the opportunity for a sustainable development approach through biomass utilization (Figure 3).

## Author Contributions

SS and MW conceptualized the topic, researched and analyzed the literature, wrote the manuscript, and including interpretations. PT analyzed the background literature and drafted portions of the manuscript. NL, KJ, PR, PS, YP, TC, WR, and PJ provided substantial scholarly guidance on the conception of the topic, manuscript draft, and interpretation and revised the manuscript critically for intellectual content. All authors contributed to the article and approved the submitted version.

## Funding

This research was partially supported by the Chiang Mai University.

## Conflict of Interest

The authors declare that the research was conducted in the absence of any commercial or financial relationships that could be construed as a potential conflict of interest.

## Publisher's Note

All claims expressed in this article are solely those of the authors and do not necessarily represent those of their affiliated organizations, or those of the publisher, the editors and the reviewers. Any product that may be evaluated in this article, or claim that may be made by its manufacturer, is not guaranteed or endorsed by the publisher.

## References

[1] Parracho H McCartney AL Gibson GR. Probiotics and prebiotics in infant nutrition. Proc Nutr Soc. (2007) 66:405–11. 10.1017/S0029665107005678 17637093

[2] Bharadia L Agrawal N Joshi N. Development and functions of the infant gut microflora: Western vs. Indian infants. Int J Pediatr. (2020) 2020:7586264. 10.1155/2020/7586264 32454840 PMC7229554

[3] Rotimi V Duerden B. The development of the bacterial flora in normal neonates. J Med Microbiol. (1981) 14:51–62. 10.1099/00222615-14-1-51 7463467

[4] Harmsen HJ Wildeboer-Veloo AC Grijpstra J Knol J Degener JE Welling GW. Development of 16S rRNA-based probes for the coriobacterium group and the Atopobium cluster and their application for enumeration of coriobacteriaceae in human feces from volunteers of different age groups. Appl Environ Microbiol. (2000) 66:4523–7. 10.1128/AEM.66.10.4523-4527.2000 11010909 PMC92335

[5] Sierra C Bernal M-J Blasco J Martínez R Dalmau J Ortuño I. Prebiotic effect during the first year of life in healthy infants fed formula containing GOS as the only prebiotic: a multicentre, randomised, double-blind and placebo-controlled trial. Eur J Nutr. (2015) 54:89–99. 10.1007/s00394-014-0689-9 24671237 PMC4303717

[6] Boehm Gn Moro G. Structural and functional aspects of prebiotics used in infant nutrition. J Nutr. (2008) 138:1818S−28. 10.1093/jn/138.9.1818S 18716193

[7] Roberfroid M Gibson GR Hoyles L McCartney AL Rastall R Rowland I. Prebiotic effects: metabolic and health benefits. Br J Nutr. (2010) 104:S1–63. 10.1017/S0007114510003363 20920376

[8] A Gosling Stevens GW Barber AR Kentish SE Gras SL. Recent advances refining galactooligosaccharide production from lactose. Food Chem. (2010) 121:307–18. 10.1016/j.foodchem.2009.12.063

[9] Rycroft CE Jones MR Gibson GR Rastall RA. A comparative in vitro evaluation of the fermentation properties of prebiotic oligosaccharides. J Appl Microbiol. (2001) 91:878–87. 10.1046/j.1365-2672.2001.01446.x 11722666

[10] Vandenplas Y Zakharova I Dmitrieva Y. Oligosaccharides in infant formula: more evidence to validate the role of prebiotics. Br J Nutr. (2015) 113:1339–44. 10.1017/S0007114515000823 25989994

[11] Kunz C Rudloff S Baier W Klein N Strobel S. Oligosaccharides in human milk: structural, functional, metabolic aspects. Annu Rev Nutr. (2000) 20:699–722. 10.1146/annurev.nutr.20.1.699 10940350

[12] Cummings JH. Short chain fatty acids in the human colon. Gut. (1981) 22:763. 10.1136/gut.22.9.763 7028579 PMC1419865

[13] Gullón B Gómez B Martínez-Sabajanes M Yáñez R Parajó JC Alonso JL. Pectic oligosaccharides: manufacture and functional properties. Trends Food Sci Technol. (2013) 30:153–61. 10.1016/j.tifs.2013.01.006

[14] Vos Av Van Esch B Stahl B M'rabet L Folkerts G Nijkamp F. Dietary supplementation with specific oligosaccharide mixtures decreases parameters of allergic asthma in mice. Int Immunopharmacol. (2007) 7:1582–7. 10.1016/j.intimp.2007.07.024 17920536

[15] Sommano S Ounamornmas P Nisoa M Sriwattana S Page P Colelli G. Characterisation and physiochemical properties of mango peel pectin extracted by conventional and phase control microwave-assisted extractions. Int Food Res J. (2018) 25:2657–65. 10.1016/j.ijbiomac.2016.06.011 27283236

[16] Wongkaew M Kittiwachana S Phuangsaijai N Tinpovong B Tiyayon C Pusadee T. Fruit characteristics, peel nutritional compositions, and their relationships with mango peel pectin quality. Plants. (2021) 10:1148. 10.3390/plants10061148 34200110 PMC8226707

[17] Chaiwarit T Masavang S Mahe J Sommano S Ruksiriwanich W Brachais C-H. Mango (cv. Nam Dokmai) peel as a source of pectin and its potential use as a film-forming polymer. Food Hydrocoll. (2020) 102:105611. 10.1016/j.foodhyd.2019.105611

[18] Wongkaew M Sommano SR Tangpao T Rachtanapun P Jantanasakulwong K. Mango peel pectin by microwave-assisted extraction and its use as fat replacement in dried Chinese sausage. Foods. (2020) 9:450. 10.3390/foods9040450 32272742 PMC7231197

[19] Wongkaew M Tinpovong B Sringarm K Leksawasdi N Jantanasakulwong K Rachtanapun P. Crude pectic oligosaccharide recovery from thai chok anan mango peel using pectinolytic enzyme hydrolysis. Foods. (2021) 10:627. 10.3390/foods10030627 33809517 PMC7999440

[20] FAO/WHO. Probiotics in food Health and nutritional properties and guidelines for evaluation. Report of a Joint FAO/WHO Expert Consultation on Evaluation of Health and Nutritional Properties of Probiotics in Food including Powder Milk with Live Lactic Acid Bacteria. Rome: FAO of The United Nation (2001).

[21] Masco L Ventura M Zink R Huys G Swings J. Polyphasic taxonomic analysis of Bifidobacterium animalis and Bifidobacterium lactis reveals relatedness at the subspecies level: reclassification of Bifidobacterium animalis as Bifidobacterium animalis subsp. animalis subsp nov and Bifidobacterium lactis as Bifidobacterium animalis subsp lactis subsp nov. Int J Syst Evol Microbiol. (2004) 54:1137–43. 10.1099/ijs.0.03011-0 15280282

[22] Holzapfel WH Haberer P Geisen R Björkroth J Schillinger U. Taxonomy and important features of probiotic microorganisms in food and nutrition. Am J Clin Nutr. (2001) 73:365–73s. 10.1093/ajcn/73.2.365s 11157343

[23] Kechagia M Basoulis D Konstantopoulou S Dimitriadi D Gyftopoulou K Skarmoutsou N. Health benefits of probiotics: a review. ISRN Nutr. (2013) 2013:481651. 10.5402/2013/481651 24959545 PMC4045285

[24] Islam SU. Clinical uses of probiotics. Medicine. (2016) 95:e2658. 10.1097/MD.0000000000002658 26844491 PMC4748908

[25] Hill C Guarner F Reid G Gibson GR Merenstein DJ Pot B. Expert consensus document. The international scientific association for probiotics and prebiotics consensus statement on the scope and appropriate use of the term probiotic. Nat Rev Gastroenterol Hepatol. (2014) 11:506–14. 10.1038/nrgastro.2014.66 24912386

[26] Plaza-Diaz J Ruiz-Ojeda FJ Gil-Campos M Gil A. Mechanisms of action of probiotics. Adv Nutr. (2019) 10:S49–66. 10.1093/advances/nmy063 30721959 PMC6363529

[27] Kandler O Stetter K-O Köhl R. Lactobacillus reuteri sp. nov, a new species of Heterofermentative Lactobacilli. Zentralblatt für Bakteriologie: I Abt Originale C: Allgemeine, angewandte und ökologische Mikrobiologie. (1980) 1:264–9. 10.1016/S0172-5564(80)80007-828820096

[28] Jacobsen CN Rosenfeldt Nielsen V Hayford AE Møller PL Michaelsen KF Paerregaard A. Screening of probiotic activities of forty-seven strains of Lactobacillus spp. by in vitro techniques and evaluation of the colonization ability of five selected strains in humans. Appl Environ Microbiol. (1999) 65:4949–56. 10.1128/AEM.65.11.4949-4956.1999 10543808 PMC91666

[29] Valeur N Engel P Carbajal N Connolly E Ladefoged K. Colonization and immunomodulation by Lactobacillus reuteri ATCC 55730 in the human gastrointestinal tract. Appl Environ Microbiol. (2004) 70:1176–81. 10.1128/AEM.70.2.1176-1181.2004 14766603 PMC348788

[30] Mu Q Tavella VJ Luo XM. Role of lactobacillus reuteri in human health and diseases. Front Microbiol. (2018) 9:757. 10.3389/fmicb.2018.00757 29725324 PMC5917019

[31] Turroni F Foroni E Pizzetti P Giubellini V Ribbera A Merusi P. Exploring the diversity of the bifidobacterial population in the human intestinal tract. Appl Environ Microbiol. (2009) 75:1534–45. 10.1128/AEM.02216-08 19168652 PMC2655441

[32] I Mainville Arcand Y Farnworth ER. A dynamic model that simulates the human upper gastrointestinal tract for the study of probiotics. Int J Food Microbiol. (2005) 99:287–96. 10.1016/j.ijfoodmicro.2004.08.020 15808363

[33] Jayamanne VS Adams MR. Determination of survival, identity and stress resistance of probiotic bifidobacteria in bio-yoghurts. Lett Appl Microbiol. (2006) 42:189–94. 10.1111/j.1472-765X.2006.01843.x 16478503

[34] Quigley EMM Chapter 13 - Bifidobacterium animalis spp. lactis. In: Floch MH, Ringel Y, Allan Walker W, editors. The Microbiota in Gastrointestinal Pathophysiology. Boston; Academic Press (2017).

[35] Larsen N Cahú TB Isay Saad SM Blennow A Jespersen L. The effect of pectins on survival of probiotic Lactobacillus spp. in gastrointestinal juices is related to their structure and physical properties. Food microbiol. (2018) 74:11–20. 10.1016/j.fm.2018.02.015 29706325

[36] Corcoran BM Stanton C Fitzgerald GF Ross RP. Survival of Probiotic Lactobacilli in Acidic Environments Is Enhanced in the Presence of Metabolizable Sugars. Appl Environ Microbiol. (2005) 71:3060–7. 10.1128/AEM.71.6.3060-3067.2005 15933002 PMC1151822

[37] Hernandez-Hernandez O Muthaiyan A Moreno FJ Montilla A Sanz ML Ricke SC. Effect of prebiotic carbohydrates on the growth and tolerance of Lactobacillus. Food Microbiol. (2012) 30:355–61. 10.1016/j.fm.2011.12.022 22365348

[38] Quigley ME Hudson GJ Englyst HN. Determination of resistant short-chain carbohydrates (non-digestible oligosaccharides) using gas–liquid chromatography. Food Chem. (1999) 65:381–90. 10.1016/S0308-8146(98)00178-2

[39] Gibson GR Roberfroid MB. Dietary modulation of the human colonic microbiota: introducing the concept of prebiotics. J Nutr. (1995) 125:1401–12. 10.1093/jn/125.6.1401 7782892

[40] Al-Sheraji SH Ismail A Manap MY Mustafa S Yusof RM Hassan FA. Prebiotics as functional foods: a review. J Funct Foods. (2013) 5:1542–53. 10.1016/j.jff.2013.08.009

[41] Gibson G Rastall R. Prebiotics: Development & Application. Wiley: West Sussex (2006).

[42] Roberfroid MB. Introducing inulin-type fructans. Br J Nutr. (2005) 93(Suppl. 1) S13–25. 10.1079/BJN20041350 15877886

[43] Ozcan O Ozcan T Yilmaz-Ersan L Akpinar-Bayizit A Delikanli B. The use of prebiotics of plant origin in functional milk products. Food Sci Technol. (2016) 4:15–22. 10.13189/fst.2016.040201

[44] Padma Ishwarya S Prabhasankar P. Prebiotics: application in bakery and pasta products. Crit Rev Food Sci Nutr. (2014) 54:511–22. 10.1080/10408398.2011.590244 24237001

[45] Eeckhaut V Van Immerseel F Dewulf J Pasmans F Haesebrouck F Ducatelle R. Arabinoxylooligosaccharides from wheat bran inhibit Salmonella colonization in broiler chickens. Poult Sci. (2008) 87:2329–34. 10.3382/ps.2008-00193 18931184

[46] Singh M Sharma R Banerjee UC. Biotechnological applications of cyclodextrins. Biotechnol Adv. (2002) 20:341–59. 10.1016/S0734-9750(02)00020-4 14550020

[47] Barczynska R Slizewska K Jochym K Kapusniak J Libudzisz Z. The tartaric acid-modified enzyme-resistant dextrin from potato starch as potential prebiotic. J Funct Foods. (2012) 4:954–62. 10.1016/j.jff.2012.07.003

[48] Sangeetha PT Ramesh MN Prapulla SG. Recent trends in the microbial production, analysis and application of Fructooligosaccharides. Trends Food Sci Technol. (2005) 16:442–57. 10.1016/j.tifs.2005.05.003

[49] Alander M Mättö J Kneifel W Johansson M Kögler B Crittenden R. Effect of galacto-oligosaccharide supplementation on human faecal microflora and on survival and persistence of Bifidobacterium lactis Bb-12 in the gastrointestinal tract. Int Dairy J. (2001) 11:17–825. 10.1016/S0958-6946(01)00100-5

[50] Lina BAR Jonker D Kozianowski G. Isomaltulose (Palatinose®): a review of biological and toxicological studies. Food Chem Toxicol. (2002) 40:1375–81. 10.1016/S0278-6915(02)00105-9 12387299

[51] Kaneko T Kohmoto T Kikuchi H Shiota M Iino H Mitsuoka T. Effects of isomaltooligosaccharides with different degrees of polymerization on human fecal bifidobactcria. Biosci Biotechnol Biochem. (1994) 58:2288–90. 10.1271/bbb.58.2288

[52] Villamiel M Corzo N Foda MI Montes F Olano AN. Lactulose formation catalysed by alkaline-substituted sepiolites in milk permeate. Food Chem. (2002) 76:7–11. 10.1016/S0308-8146(01)00239-4

[53] Babbar N Dejonghe W Sforza S Elst K. Enzymatic pectic oligosaccharides (POS) production from sugar beet pulp using response surface methodology. J Food Sci Technol. (2017) 54:3707–15. 10.1007/s13197-017-2835-x 29051666 PMC5629180

[54] Zhang S Hu H Wang L Liu F Pan S. Preparation and prebiotic potential of pectin oligosaccharides obtained from citrus peel pectin. Food Chem. (2018) 244:232–7. 10.1016/j.foodchem.2017.10.071 29120775

[55] Johansen HN Glitsø V Bach Knudsen KE. Influence of extraction solvent and temperature on the quantitative determination of oligosaccharides from plant materials by high-performance liquid chromatography. J Agric Food Chem. (1996) 44:470–1474. 10.1021/jf950482b

[56] Mussatto SI Mancilha IM. Non-digestible oligosaccharides: a review. Carbohydr Polym. (2007) 68:587–97. 10.1016/j.carbpol.2006.12.011

[57] Vázquez MJ Alonso JL Dominguez H Parajó JC. Xylooligosaccharides: manufacture and applications. Trends Food Sci Technol. (2000) 11:387–93. 10.1016/S0924-2244(01)00031-0

[58] González-Herrera SM Herrera RR López MG Rutiaga OM Aguilar CN Esquivel JCC. Inulin in food products: prebiotic and functional ingredient. Br Food J. (2015) 117:371–8. 10.1108/BFJ-09-2013-023834742431

[59] Míguez B Gómez B Gullón P Gullón B Alonso JL. Pectic oligosaccharides and other emerging prebiotics. Probiotics and Prebiotics in Human Nutrition and Health. London: IntechOpen Limited (2016).

[60] Garthoff JA Heemskerk S Hempenius RA Lina BA Krul CA Koeman JH. Safety evaluation of pectin-derived acidic oligosaccharides (pAOS): genotoxicity and sub-chronic studies. Regul Toxicol Pharmacol. (2010) 57:31–42. 10.1016/j.yrtph.2009.12.004 20026148

[61] Baldan B Bertoldo A Navazio L Mariani P. Oligogalacturonide-induced changes in the developmental pattern of Daucus carota L. somatic embryos. Plant Sci. (2003) 165:337–48. 10.1016/S0168-9452(03)00193-6

[62] Manderson K Pinart M Tuohy KM Grace WE Hotchkiss AT Widmer W. *In vitro* determination of prebiotic properties of oligosaccharides derived from an orange juice manufacturing by-product stream. Appl Environ Microbiol. (2005) 71:8383–9. 10.1128/AEM.71.12.8383-8389.2005 16332825 PMC1317361

[63] Chen J Liang RH Liu W Li T Liu CM Wu SS. Pectic-oligosaccharides prepared by dynamic high-pressure microfluidization and their in vitro fermentation properties. Carbohydr Polym. (2013) 91:175–82. 10.1016/j.carbpol.2012.08.021 23044120

[64] Combo AMM Aguedo M Goffin D Wathelet B Paquot M. Enzymatic production of pectic oligosaccharides from polygalacturonic acid with commercial pectinase preparations. Food Bioprod Process. (2012) 90:588–96. 10.1016/j.fbp.2011.09.003

[65] A Wilkowska Nowak A Antczak-Chrobot A Motyl I Czyzowska A Paliwoda A. Structurally different pectic oligosaccharides produced from apple pomace and their biological activity *in vitro*. Foods. (2019) 8:365. 10.3390/foods8090365 31454989 PMC6769907

[66] Sabater A Blanco-Doval A Montilla A Corzo N. Optimisation of an enzymatic method to obtain modified artichoke pectin and pectic oligosaccharides using artificial neural network tools. In silico and *in vitro* assessment of the antioxidant activity. Food Hydrocoll. (2021) 110:106161. 10.1016/j.foodhyd.2020.106161

[67] Ho Y-Y Lin C-M Wu M-C. Evaluation of the prebiotic effects of citrus pectin hydrolysate. J Food Drug Anal. (2017) 25:550–8. 10.1016/j.jfda.2016.11.014 28911641 PMC9328821

[68] A Gómez Gullón B Yáñez R Schols H Alonso JL. Prebiotic potential of pectins and pectic oligosaccharides derived from lemon peel wastes and sugar beet pulp: a comparative evaluation. J Funct Foods. (2016) 20:108–21. 10.1016/j.jff.2015.10.029

[69] Li P-J Xia J-l Nie Z-Y Shan Y. Pectic oligosaccharides hydrolyzed from orange peel by fungal multi-enzyme complexes and their prebiotic and antibacterial potentials. LWT Food Sci Technol. (2016) 69:203–10. 10.1016/j.lwt.2016.01.042

[70] Ferreira-Lazarte A Kachrimanidou V Villamiel M Rastall RA Moreno FJ. *In vitro* fermentation properties of pectins and enzymatic-modified pectins obtained from different renewable bioresources. Carbohydr Polym. (2018) 199:482–91. 10.1016/j.carbpol.2018.07.041 30143153

[71] Gómez B Gullón B Yáñez R Parajó JC Alonso JL. Pectic oligosacharides from lemon peel wastes: production, purification, chemical characterization. J Agric Food Chem. (2013) 61:10043–53. 10.1021/jf402559p 24066740

[72] Zhu R Wang C Zhang L Wang Y Chen G Fan J. Pectin oligosaccharides from fruit of actinidia arguta: structure-activity relationship of prebiotic and antiglycation potentials. Carbohydr Polym. (2019) 217:90–7. 10.1016/j.carbpol.2019.04.032 31079689

[73] Zhu R Zhang X Wang Y Zhang L Wang C Hu F. Pectin oligosaccharides from hawthorn (Crataegus pinnatifida Bunge. Var major): molecular characterization and potential antiglycation activities. Food Chem. (2019) 286:129–35. 10.1016/j.foodchem.2019.01.215 30827585

[74] Babbar N Dejonghe W Gatti M Sforza S Elst K. Pectic oligosaccharides from agricultural by-products: production, characterization and health benefits. Crit Rev Biotechnol. (2016) 36:594–606. 10.3109/07388551.2014.996732 25641325

[75] Kim S-K Rajapakse N. Enzymatic production and biological activities of chitosan oligosaccharides (COS): a review. Carbohydr Polym. (2005) 62:357–68. 10.1016/j.carbpol.2005.08.012

[76] Concha Olmos J Zúñiga Hansen ME. Enzymatic depolymerization of sugar beet pulp: Production and characterization of pectin and pectic-oligosaccharides as a potential source for functional carbohydrates. Chem Eng J. (2012) 192:29–36. 10.1016/j.cej.2012.03.085

[77] Pedrolli D Monteiro A Gomes E Carmona E. Pectin and pectinases: production, characterization and industrial application of microbial pectinolytic enzymes. Open Biotechnol J. (2009) 3:9–18. 10.2174/1874070700903010009

[78] Shevchik VE Hugouvieux-Cotte-Pattat N. Identification of a bacterial pectin acetyl esterase in Erwinia chrysanthemi 3937. Mol Microbiol. (1997) 24:1285–301. 10.1046/j.1365-2958.1997.4331800.x 9218776

[79] Cameron R Luzio G Savary B Goodner K. Digestion patterns of two commercial endopolygalacturonases on polygalacturonate oligomers with a degree of polymerization of 7 to 21. Proc Fla State Hortic Soc. (2009) 122:295–302.

[80] Parenicová L Benen JA Kester HC Visser J. pgaA and pgaB encode two constitutively expressed endopolygalacturonases of Aspergillus niger. Biochem J. (2000) 345:637–44. 10.1042/bj3450637 10642523 PMC1220799

[81] Pedrolli DB Carmona EC. Purification and characterization of a unique pectin lyase from *Aspergillus giganteus* able to release unsaturated monogalacturonate during pectin degradation. Enzyme Res. (2014) 2014:353915. 10.1155/2014/353915 25610636 PMC4294307

[82] Martínez M Gullón B Schols HA Alonso JL Parajó JC. Assessment of the production of oligomeric compounds from sugarbeet pulp. Ind Eng Chem Res. (2009) 48:4681. 10.1021/ie8017753

[83] Lama-Muñoz A Rodríguez-Gutiérrez G Rubio-Senent F Fernández-Bolaños J. Production, characterization and isolation of neutral and pectic oligosaccharides with low molecular weights from olive by-products thermally treated. Food Hydrocoll. (2012) 28:92–104. 10.1016/j.foodhyd.2011.11.008

[84] Olano-Martin A Gibson GR Rastell RA. Comparison of the in vitro bifidogenic properties of pectins and pectic-oligosaccharides. J Appl Microbiol. (2002) 93:505–11. 10.1046/j.1365-2672.2002.01719.x 12174051

[85] Ignatova T Iliev I Kirilov N Vassileva T Dalgalarrondo M Haertle T. Effect of oligosaccharides on the growth of Lactobacillus delbrueckii subsp. bulgaricus strains isolated from dairy products. J Agric Food Chem. (2009) 57:9496–502. 10.1021/jf901684z 20560621

[86] Holck J Lorentzen A Vigsnæs LK Licht TR Mikkelsen JD Meyer AS. Feruloylated and nonferuloylated arabino-oligosaccharides from sugar beet pectin selectively stimulate the growth of Bifidobacterium spp. in human fecal in vitro fermentations. J Agric Food Chem. (2011) 59:6511–9. 10.1021/jf200996h 21574556

[87] Garna H Mabon N Nott K Wathelet B Paquot M. Kinetic of the hydrolysis of pectin galacturonic acid chains and quantification by ionic chromatography. Food Chem. (2006) 96:477–84. 10.1016/j.foodchem.2005.03.002

[88] Leijdekkers A Bink J Geutjes S Schols HA Gruppen H. Enzymatic saccharification of sugar beet pulp for the production of galacturonic acid and arabinose, a study on the impact of the formation of recalcitrant oligosaccharides. Bioresour Technol. (2012) 128C:518–25. 10.1016/j.biortech.2012.10.126 23202377

[89] Gómez A Gullón B Remoroza C Schols HA Parajó JC Alonso JL. Purification, characterization, and prebiotic properties of pectic oligosaccharides from orange peel wastes. J Agric Food Chem. (2014) 62:9769–82. 10.1021/jf503475b 25207862

[90] Holck J Hjernø K Lorentzen A Vigsnæs LK Hemmingsen L Licht TR. Tailored enzymatic production of oligosaccharides from sugar beet pectin and evidence of differential effects of a single DP chain length difference on human faecal microbiota composition after *in vitro* fermentation. Process Biochem. (2011) 46:1039–49. 10.1016/j.procbio.2011.01.013

[91] Iwasaki K-I Matsubara Y. Purification of pectate oligosaccharides showing root-growth-promoting activity in lettuce using ultrafiltration and nanofiltration membranes. J Biosci Bioeng. (2000) 89:495–7. 10.1016/S1389-1723(00)89104-5 16232785

[92] Guillaumie A Justesen SF Mutenda KE Roepstorff P Jensen KJ Thomas OR. Fractionation, solid-phase immobilization and chemical degradation of long pectin oligogalacturonides. Initial steps towards sequencing of oligosaccharides. Carbohydr Res. (2006) 341:118–29. 10.1016/j.carres.2005.10.011 16297890

[93] Ralet M-C Cabrera JC Bonnin E Quéméner B Hellìn P Thibault J-F. Mapping sugar beet pectin acetylation pattern. Phytochemistry. (2005) 66:1832–43. 10.1016/j.phytochem.2005.06.003 16024056

[94] Coenen GJ Kabel MA Schols HA Voragen AG. CE-MSn of complex pectin-derived oligomers. Electrophoresis. (2008) 29:2101–11. 10.1002/elps.200700465 18425747

[95] Ström A Williams MA. On the separation, detection and quantification of pectin derived oligosaccharides by capillary electrophoresis. Carbohydr Res. (2004) 339:1711–6. 10.1016/j.carres.2004.05.010 15220080

[96] Ralet MC Lerouge P Quéméner B. Mass spectrometry for pectin structure analysis. Carbohydr Res. (2009) 34:1798–807. 10.1016/j.carres.2008.08.036 19058795

[97] Colquhoun IJ de Ruiter GA Schols HA Voragen AG. Identification by nmr spectroscopy of oligosaccharides obtained by treatment of the hairy regions of apple pectin with rhamnogalacturonase. Carbohydr Res. (1990) 206:131–44. 10.1016/0008-6215(90)84012-J 2081341

[98] Renard CM Lahaye M Mutter M Voragen FG Thibault JF. Isolation and structural characterisation of rhamnogalacturonan oligomers generated by controlled acid hydrolysis of sugar-beet pulp. Carbohydr Res. (1997) 305:271–80. 10.1016/S0008-6215(97)10028-3 9581279

[99] Ishii T Ichita J Matsue H Ono H Maeda I. Fluorescent labeling of pectic oligosaccharides with 2-aminobenzamide and enzyme assay for pectin. Carbohydr Res. (2002) 337:1023–32. 10.1016/S0008-6215(02)00087-3 12039543

[100] Westphal Y Kühnel S Schols HA Voragen AG Gruppen H. LC/CE-MS tools for the analysis of complex arabino-oligosaccharides. Carbohydr Res. (2010) 345:2239–51. 10.1016/j.carres.2010.07.011 20732678

[101] Combo AMM Aguedo M Quiévy N Danthine S Goffin D Jacquet N. Characterization of sugar beet pectic-derived oligosaccharides obtained by enzymatic hydrolysis. Int J Biol Macromol. (2013) 52:148–56. 10.1016/j.ijbiomac.2012.09.006 22986181

[102] Remoroza C Buchholt HC Gruppen H Schols HA. Descriptive parameters for revealing substitution patterns of sugar beet pectins using pectolytic enzymes. Carbohydr Polym. (2014) 101:1205–15. 10.1016/j.carbpol.2013.10.034 24299893

[103] Barrangou R Altermann E Hutkins R Cano R Klaenhammer T. Functional and comparative genomic analyses of an operon involved in fructooligosaccharide utilization by Lactobacillus acidophilus. Proc Natl Acad Sci U S A. (2003) 100:8957–62. 10.1073/pnas.1332765100 12847288 PMC166420

[104] Mueller I Kiedorf G Runne E Seidel-Morgenstern A Hamel Synthesis C. Kinetic analysis and modelling of galacto-oligosaccharides formation. Chem Eng Res Des. (2017) 130:154–66. 10.1016/j.cherd.2017.11.03820681989

[105] Burana-Osot J Soonthornchareonnon N Chaidedgumjorn A Hosoyama S Toida T. Determination of galacturonic acid from pomelo pectin in term of galactose by HPAEC with fluorescence detection. Carbohydr Polym. (2010) 81:461. 10.1016/j.carbpol.2010.03.001

[106] Kang HJ Jo C Kwon JH Son JH An BJ Byun MW. Antioxidant and cancer cell proliferation inhibition effect of citrus pectin-oligosaccharide prepared by irradiation. J Med Food. (2006) 9:313–20. 10.1089/jmf.2006.9.313 17004892

[107] Li T Li S Du L Wang N Guo M Zhang J. Effects of haw pectic oligosaccharide on lipid metabolism and oxidative stress in experimental hyperlipidemia mice induced by high-fat diet. Food Chem. (2010) 121:1010–3. 10.1016/j.foodchem.2010.01.039

[108] Hotchkiss A Olano-Martin E Grace W Gibson G Rastall R. Pectic Oligosaccharides as Prebiotics. Cambridge: Society for Applied Microbiology Wiley (2003).

[109] Dongowski A Lorenz A. Unsaturated oligogalacturonic acids are generated by *in vitro* treatment of pectin with human faecal flora. Carbohydr Res. (1998) 314:237–44. 10.1016/S0008-6215(98)00304-8 10335591

[110] Olano-Martin E Williams MR Gibson GR Rastall RA. Pectins and pectic-oligosaccharides inhibit Escherichia coli O157:H7 Shiga toxin as directed towards the human colonic cell line HT29. FEMS Microbiol Lett. (2003) 218:101–5. 10.1111/j.1574-6968.2003.tb11504.x 12583904

[111] Rhoades J Manderson K Wells A Hotchkiss A Gibson G Formentin K. Oligosaccharide-mediated inhibition of the adhesion of pathogenic Escherichia coli strains to human gut epithelial cells *in vitro*. J Food Prot. (2008) 71:2272–7. 10.4315/0362-028X-71.11.2272 19044272

[112] Zhang A Zhang M Tao Y Wang G Xia B. Madecassic acid inhibits the mouse colon cancer growth by inducing apoptosis and immunomodulation. J BUON. (2014) 19:372–6. 24965394

[113] Vulevic J Drakoularakou A Yaqoob P Tzortzis G Gibson GR. Modulation of the fecal microflora profile and immune function by a novel trans-galactooligosaccharide mixture (B-GOS) in healthy elderly volunteers. Am J Clin Nutr. (2008) 88:1438–46. 10.3945/ajcn.2008.26242 18996881

[114] Vos AP Haarman M van Ginkel JW Knol J Garssen J Stahl B. Dietary supplementation of neutral and acidic oligosaccharides enhances Th1-dependent vaccination responses in mice. Pediatr Allergy Immunol. (2007) 18:304–12. 10.1111/j.1399-3038.2007.00515.x 17584310

[115] Gullón B Gullón P Sanz Y Alonso JL Parajó JC. Prebiotic potential of a refined product containing pectic oligosaccharides. LWT Food Sci Technol. (2011) 44:1687–96. 10.1016/j.lwt.2011.03.00625207862

[116] Chung WSF Meijerink M Zeuner B Holck J Louis P Meyer AS. Prebiotic potential of pectin and pectic oligosaccharides to promote anti-inflammatory commensal bacteria in the human colon. FEMS Microbiol Ecol. (2017) 93:fix127. 10.1093/femsec/fix127 29029078

[117] Hotchkiss AT Olano-Martin E Grace WE Gibson GR Rastall RA. Pectic Oligosaccharides as Prebiotics, Oligosaccharides in Food and Agriculture, American Chemical Society. Washington, DC: American Chemical Society (2003).

[118] Al-Tamimi AHM Palframan RJ Cooper JM Gibson GR Rastall RA. *In vitro* fermentation of sugar beet arabinan and arabino-oligosaccharides by the human gut microflora. J Appl Microbiol. (2006) 100:407–14. 10.1111/j.1365-2672.2005.02780.x 16430518

[119] Yeung YK Kang Y-R So BR Jung SK Chang YH. Structural, antioxidant, prebiotic and anti-inflammatory properties of pectic oligosaccharides hydrolyzed from okra pectin by Fenton reaction. Food Hydrocoll. (2021) 118:106779. 10.1016/j.foodhyd.2021.106779

[120] Mao X Xiao X Chen D Yu B He J Chen H. Dietary apple pectic oligosaccharide improves gut barrier function of rotavirus-challenged weaned pigs by increasing antioxidant capacity of enterocytes. Oncotarget. (2017) 8:92420–30. 10.18632/oncotarget.21367 29190927 PMC5696193

[121] Xue L Long J Lu C Li X Xu X Jin Z. Immobilization of polygalacturonase for the preparation of pectic oligosaccharides from mango peel wastes and assessment of their antibacterial activities. Food Biosci. (2021) 39:100837. 10.1016/j.fbio.2020.100837

